# Sarcopenic Obesity and Cognitive Functioning: The Mediating Roles of Insulin Resistance and Inflammation?

**DOI:** 10.1155/2012/826398

**Published:** 2012-05-07

**Authors:** M. E. Levine, E. M. Crimmins

**Affiliations:** Davis School of Gerontology, University of Southern California, Los Angeles, CA 90089-0191, USA

## Abstract

This study examined the influence of insulin resistance and inflammation on the association between body composition and cognitive performance in older adults, aged 60–69 and aged 70 and older. Subjects included 1127 adults from NHANES 1999–2002. Body composition was categorized based on measurements of muscle mass and waist circumference as sarcopenic nonobese, nonsarcopenic obese, sarcopenic obese, and normal. Using OLS regression models, our findings suggest body composition is not associated with cognitive functioning in adults ages 60–69; however, for adults aged 70 and over, sarcopenia and obesity, either independently or concurrently, were associated with worse cognitive functioning relative to non-sarcopenic non-obese older adults. Furthermore, insulin resistance accounted for a significant proportion of the relationship between cognitive performance and obesity, with or without sarcopenia. Additionally, although high CRP was significantly associated with poorer cognitive functioning in adults ages 60–69, it did not influence the association between body composition and cognitive performance. This study provides evidence that age-related physiological maladaptations, such as metabolic deregulation, which are associated with abdominal fat, may simultaneously contribute to lower cognition and muscle mass, reflecting a degradation of multiple physiological systems.

## 1. Introduction

Both obesity and frailty or underweight status have been identified as important risk factors for poor cognitive functioning among older adults. White matter lesions and cerebral atrophy have been found to be more common in adults with a high body mass index (BMI) [1, 2], and midlife measures of central obesity have been found to predict poor performance on tests measuring executive functioning and visuomotor skills [3]. It is also true that being underweight or frail may be linked to cognitive performance. Sturman et al. found a curvilinear association between BMI and cognition at baseline; however, after 6 years, subjects who were underweight experienced greater cognitive decline than normal weight subjects, while obese subjects did not have significant declines in cognition [4]. Given that the impact of body composition is not fully understood, more research is needed to further uncover the mechanisms driving the association. Furthermore, little information currently exists regarding the confluence of body composition phenotypes and the physiological mechanisms which directly or indirectly affect cognitive performance.

As individuals age, increases in fat mass are hypothesized to stem from reductions in physical activity and retained caloric intake levels in the presence of a declining basal metabolic rate [5]. Concurrently, many individuals also experience significant declines in muscle mass and strength with aging, referred to as sarcopenia [6–8]. Age-related changes in muscle mass and adiposity may stem from a mutual pathophysiological mechanism. It has been proposed that systemic inflammation and insulin resistance may play a part in the concurrence of abdominal obesity and sarcopenia. Proinflammatory cytokines are synthesized and secreted by adipocytes [9] and may promote the breakdown of skeletal muscle fiber diameter and protein content, as well as disrupt muscle force production and fatigue resistance [10]. Additionally, insulin resistance, which is often observed in subjects with excess visceral adiposity, due to increases in cytokine production [11], is believed to contribute to metabolic deterioration of skeletal muscle, manifesting clinically as sarcopenia [12].

Age-related alterations in inflammation and insulin resistance may also have implications for cognitive functioning and may partially explain the associations between poor cognitive performance and body composition. Systemic inflammation has been identified as a potential risk factor for cognitive impairment. In cross-sectional studies, increased levels of CRP were associated with lower levels of executive functioning and global cognition [13]. Furthermore, longitudinal studies have provided evidence that high levels of CRP and IL-6 may accelerate the rate of cognitive impairment for high functioning older adults [14]. Poor cognitive functioning has also been associated with disruptions in metabolic processing. The importance of the insulin-signaling pathway has been identified as a factor facilitating the maintenance of cognitive functioning ability. Furthermore, higher insulin resistance, as measured using the homeostasis model of assessment (IR_HOMA_), has been found to be associated with poorer cognitive efficiency and poorer visual scanning with added cognitive flexibility [15].

Insulin resistance, inflammation, cognitive functioning, and body composition are strongly linked to aging and may represent an age-related decline in multiple physiological systems. Our study examines a nationally representative sample of community-dwelling US citizens over the age of 60 to determine whether insulin resistance and inflammation partially account for the associations between cognitive functioning and body composition. Because the variables being examined are closely linked to age [16, 17], our analysis is run for two separate cohorts, in order to investigate whether the influence is different at various stages of old age. Furthermore, to more precisely study the association between cognitive performance and a diverse spectrum of body types related to changes with age, we examine body composition as a combination of abdominal adiposity and muscle mass to facilitate our ability to examine an interaction between obesity and frailty. Based on previous research, we hypothesize that individuals who are sarcopenic obese will have lower cognitive ability than nonsarcopenic nonobese subjects and that this association will be explained, in part, by high levels of insulin resistance and CRP. Furthermore, because cognitive functioning, body composition, and physiological health deteriorate with age, we hypothesize that these associations will be stronger among subjects aged 70 and over, as compared to subjects aged 60–69.

## 2. Materials and Methods

### 2.1. Study Population

The study population was comprised of males and females aged 60 and older from two subsamples of the US National Health and Nutrition Examination Survey (NHANES 1999–2002)—a nationally representative, cross-sectional study conducted by the National Center for Health Statistics (NCHS) [18]. Data were collected during at-home interviews and at clinical and laboratory examinations, taking place at a Mobile Examination Center (MEC). Anthropometric data measurements for body composition were measured by dual X-ray adsorptiometry (DXA). Because of the potential for nonresponse bias in the use of only cases with information from the DXA scans, imputed data were utilized in analysis as developed and recommended by NHANES [18]. The NHANES files contain five sets of imputed data for each eligible participant with missing DXA data. While only one record was used in calculating sample sizes, all five records were used in analyses to insure more accurate variance estimates were obtained.

The total subsample size for eligible participants over age 60 from both NHANES 1999-2000 and 2001-2002 was 1630. Due to missing data from the examination and laboratory tests, those who were included in the study were 70% (*N* = 1127) of the original subsample, of whom 555 were aged 60–69 and 572 were aged 70 and over. The subsample included individuals who were tested for plasma glucose in a morning laboratory exam and who had fasted for eight to twelve hours before. Because individuals with diagnosed diabetes were not asked to fast, those individuals were excluded from the study. Further details of recruitment, procedures, and study design are available through the U.S. Department of Health and Human Services [18].

### 2.2. Cognitive Functioning

 Cognitive functioning was based on the score for the Wechsler Adult Intelligence Scale, Third Edition (WAIS-III) Digit Symbol-Coding module administered during the household interview. Scores are based on the number of correctly drawn symbols, out of 133, within a 120-second period. Subjects who were unable to complete the cognitive performance task without assistance were not given scores and, therefore, not included in our analysis. The scale is considered to be a more precise indicator of diminished cognitive skills than the Minimental Status Exam and has been administered in the Health ABC study from the National Institute on Aging [18].

### 2.3. Body Composition

 Body composition measurements included waist circumference, weight, height, and skeletal muscle mass (SMM). Body weight was measured using standardized procedures and equipment and was recorded to the nearest 0.01 kg by electronic scale. Waist circumference was measured to the nearest 0.1 cm starting on the right side of the body at the iliac crest. Measurements for regional bone, fat, and lean-tissue content were collected by whole body DXA scans. We estimated SMM using measurements of total lean muscle mass. Lean mass was estimated from DXA measurements in kilograms. Subjects with an SMM less than one standard deviation below the mean of a young reference group, which included males and females ages 20–39 from NHANES 1999–2003, were classified as sarcopenic. The cutoffs derived from the reference group were 48.37 kg for males and 34.57 kg for females. Waist circumference was used to define obesity given that it is less likely to be confounded with muscle mass than is body mass index (BMI). Furthermore, given that insulin resistance and inflammation are more closely associated with abdominal adiposity in comparison with other types of fat, we chose to use waist circumference given its ability to predict abdominal tissue mass located in the midsection. Obesity was defined as waist circumference greater than 102 cm for males and 88 cm for females [19]. Because our obesity and sarcopenia measures may be confounded by height, we included a measure of standing height (in cm) as a control during analysis. Based on these cutoffs, four categorical groups were created—solely sarcopenic, solely obese, sarcopenic obese, and normal. Finally, although there is no cutoff for the lower end of waist circumference, 95.5% of subjects who would have been classified as underweight using BMI (<18.5) were captured in the sarcopenic nonobese group while the remaining 5% were in the reference group due to their healthy levels of muscle mass.

### 2.4. Insulin Resistance and Inflammation

 Insulin resistance was determined through the use of the homeostasis model assessment (IR_HOMA_) [20]. IR_HOMA_ is relevant to epidemiological studies and facilitates the estimation of insulin resistance using plasma glucose and insulin. It has also been shown to closely correlate with the insulin sensitivity index [21]. IR_HOMA_ was calculated as the product of fasting glucose (mmol/L) and fasting insulin (*μ*U/mL) divided by 22.5 [22]. CRP in a nonspecific indicator of general levels of systemic inflammation. In NHANES 1999–2002, high-sensitivity CRP assays were performed on blood samples using a Behring Nephelometer for quantitative CRP determination [18].

### 2.5. Potential Confounders

 Age, sex, race/ethnicity, education, low physical activity level, and history of cardiovascular disease (CVD) were collected via self-report during the interview. In analysis, these were used as control variables as they have been found to relate to obesity, sarcopenia, and cognitive decline [23–29]. In the NHANES data, age was measured as a continuous variable, in years, and top-coded at 85 in order to guarantee anonymity among the oldest-old sample. Because cognitive functioning, adiposity, muscle mass, systemic inflammation, and insulin sensitivity are strongly linked to aging, our analysis is performed separately for the younger part of the sample, those aged 60–69, and those aged 70 and over. In our analysis for the younger group, a continuous measure of age was used as a control; however, due to top coding in NHANES, four age groups—70–74 years, 75–79 years, 80–84 years, and ages 85+ years—were used in the 70 and over group. Four categories for race/ethnicity were constructed using dummy variables to classify participants as non-Hispanic whites, non-Hispanic blacks, Hispanic, or other, with non-Hispanic whites used as a reference category. Education was assessed as years of school completed. Low physical activity level was assessed by asking subjects to rate their average physical activity level each day. Those who reported that they sit and do not walk around very much were coded as 1, while all others were coded as 0. CVD was coded as 1 in subjects reporting ever being diagnosed with one of the following: coronary heart disease, congestive heart failure, myocardial infarction, or stroke; all others were coded as 0.

Two indicators for poor nutritional status and dietary deficiencies, hyperhomocysteinemia and hypoalbuminemia, were also used as controls. These indicators have been found to vary by age and relate closely to both body composition and cognitive performance. Homocysteine is an amino acid that at elevated levels may signal nutritional deficiencies. Total plasma homocysteine was measured using a fluorescence polarization immunoassay (FPIA) from Abbott Diagnostics, “Abbott Homocysteine (HCY) assay.” A cutoff of 15 *μ*mol/L [30] was used to classify hyperhomocysteinemia. Serum Albumin, a commonly utilized marker of nutritional status [31], was measured using Boehringer Mannheim Diagnostics. The Bromocresol purple binds selectively with albumin, at the reaction pH [17]. Hypoalbuminemia was defined as serum albumin measures less than <3.8 g/dL. Although the typical cutoff for hypoalbuminemia is reported as 3.5 g/dL [32], it has been suggested that among outpatients this threshold may be too selective and could potentially miss substantially at risk older adults [33]. Thus, a more modest hypoalbuminemia level has been suggested for use in population studies [34].

### 2.6. Statistical Analysis

 SAS statistical software package version 9.2 was used for all analyses. All analyses were run accounting for the complex sampling procedures in NHANES [20]. CRP and IR_HOMA_ were log-transformed to give them a more normal distribution and to better satisfy the assumptions of linear regression. Mean comparisons for cognitive functioning, log-transformed IR_HOMA_, and log-transformed CRP were examined by age category and body composition, using a Bonferroni adjustment. A series of three ordinary least squares regression models were run for each age group to determine whether insulin resistance and/or inflammation impacted the association between body composition and cognitive functioning. The first model was used to determine the association between body composition and cognitive functioning, adjusting for demographic, health, and nutrition variables, including age, race/ethnicity, sex, education, height, history of CVD, physical activity, hypoalbuminemia, and hyperhomocysteinemia. In models two and three, log-transformed IR_HOMA_ and log-transformed CRP were added to the original model respectively.

## 3. Results

Demographic, physical, and examination characteristics of the sample are listed by age group in Table 1. Approximately, 49.01% of subjects were 60–69 years of age with a median age of 64, while 50.99% were 70 years of age and older with a median age of 76. Participants were 53.63% and 60.23% female in the younger and older age groups, respectively. Both groups were predominately non-Hispanic white, 78.33% in the younger age group and 83.16% in the older age group. The median education for both groups was 12 years. In the younger group, 23.51% had low daily physical activity, 15.25% had CVD, 3.83 had hypoalbuminemia, and 4.71% had hyperhomocysteinemia. In the older age group, these percentages increased to 30.76% with low daily physical activity, 24.70% with CVD, 5.11% with hypoalbuminemia, and 11.20% with hyperhomocysteinemia.

**Figure 1:**
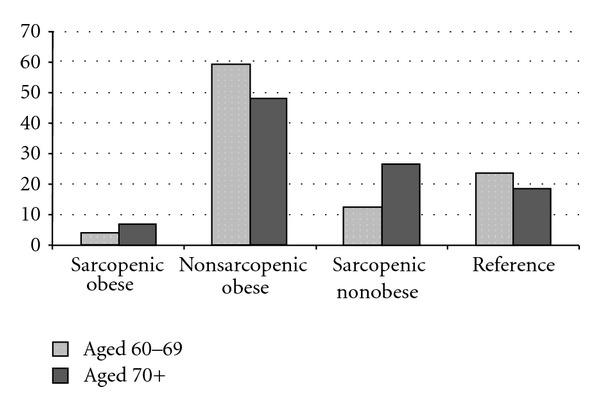
Distributions (%) of body composition by age.

Distributions of body composition, by age, are shown in Figure 1. In the 60–69-year age group, 4.40% of the subjects met criteria for sarcopenic obesity, 59.17% were solely obese, 12.63% were solely sarcopenic, and the remainder (23.80%) were neither obese nor sarcopenic. In the 70 years and over group, 7.04% of the subjects met criteria for sarcopenic obesity, 48.12% were solely obese, 26.53% were solely sarcopenic, and the remainder (18.30%) were neither obese nor sarcopenic.

Means for cognitive functioning scores and medians for IR_HOMA_ and CRP are provided by body composition and age in Table 2. For both age groups, nonsarcopenic obese subjects had significantly higher IR_HOMA_, followed by sarcopenic obese subjects and, finally, sarcopenic nonobese subjects and nonsarcopenic nonobese subjects, who had statistically comparable levels of IR_HOMA_. In both age groups, cognitive functioning scores and CRP differed significantly across all four body composition categories. Cognitive functioning was lowest among the sarcopenic obese group, followed by the sarcopenic nonobese group, nonsarcopenic obese group, and reference group. Sarcopenic obese subjects also had the highest levels of CRP, followed by subjects in the nonsarcopenic obese, sarcopenic nonobese, and the reference groups.

Table 3 examines the results from the three ordinary least squares regression models for the 60–69 age group. None of the unhealthy body composition groups were found to be significantly different from the healthy reference group in cognitive functioning score in any of the three models. Insulin resistance was not significantly related to cognitive functioning in this age group. On the other hand, CRP was found to be negatively associated with cognitive functioning. In model 1, 47.2% of the variance in cognitive functioning was explained by the variables in the equation. This is not increased in either of the subsequent models.

Table 4 shows the results from the three ordinary least squares regression models for the 70 and over age group. In the first model, all three sarcopenic and/or obese body composition groups were associated with poorer cognitive functioning. Relative to the healthy reference group, being sarcopenic obese was associated with an estimated 7-point decrease in cognitive functioning scores (*β* = −7.08, *P* < .0001), while being solely sarcopenic or solely obese was associated with estimated decreases of approximately 4.2 and 1.5 points, respectively (*β*_sarcopenic  nonobese_ = −4.19, *P* < .0001; *β*_nonsarcopenic  obese_ = −1.43, *P* < .0001). In model 2, insulin resistance was found to have a significant negative association with cognitive functioning (*β* = −3.02, *P* < .0001). The inclusion of log-transformed IR_HOMA_ in the model reduced the power of sarcopenic obesity to predict cognitive functioning by over 20% (*β* = −5.66, <.0001). Controlling for insulin resistance also eliminated the association between cognitive functioning and nonsarcopenic obesity (*β* = −0.51, *P* = .489). In model 3, CRP was not found to be a significant predictor of cognitive functioning. Furthermore, with the inclusion of CRP, the power of sarcopenic obesity, sarcopenic nonobesity, or nonsarcopenic obesity to predict cognitive functioning was not significantly altered.

## 4. Discussion

 Our findings suggest that body composition did not predict cognitive functioning in adults ages 60–69; however, for adults aged 70 and over, sarcopenia and obesity, either independently or concurrently, are associated with lower cognitive functioning when compared to nonsarcopenic nonobese older adults. Furthermore, we found that insulin resistance may account for a significant proportion of the relationship between cognitive performance and obesity, with or without sarcopenia. These results are consistent with findings that obesity, poor muscle quality, and insulin resistance [1–4, 15, 35] are associated with decreased cognitive functioning.

Age-related physiological maladaptations, such as metabolic deregulation, which are associated with abdominal fat, may simultaneously contribute to lower cognition and muscle mass, reflecting a degradation of multiple physiological systems. Insulin resistance, which often occurs as a result of the presence of excess visceral adiposity which increases with age, has been shown to alter lipid metabolism, increase systemic inflammation, disrupt endothelial functioning, and impact prothrombotic status and atherosclerosis [36]. As a result, many age-related diseases have been attributed to the steady increase in insulin resistance over the lifespan [36]. The association between insulin resistance and poor cognitive functioning may involve insulin-degrading enzyme (IDE), which plays a role in both insulin and *β*-amyloid metabolism. The accumulation of *β*-amyloid in the brain is considered one of the earliest detectable signs in the progression of Alzheimer's disease and is associated with cognitive decline, neurodegeneration, and synaptic dysfunction [37]. In the presence of insulin resistance, excess circulating insulin may prompt an increase in *β*-amyloid due to their competing demands for IDE [38].

Similarly, muscle weakness and deterioration, loss of lower extremity mobility, and changes in body composition, can also be linked to alterations in insulin sensitivity [39, 40]. It has been suggested that muscle metabolism may play a role in the development of sarcopenia, given that impaired insulin sensitivity has been shown to disrupt the anabolic effects on muscle proteins necessary for skeletal muscle conservation [40, 41]. As a result obese individuals with high levels of insulin resistance may be at increased risk of sarcopenia. On the other hand, the confluence of sarcopenia with obesity may have an even greater effect on insulin sensitivity, given that reduced muscle mass decreases the availability of insulin-responsive target tissue [42].

There are limitations in the present study which should be noted. First, the use of cross-sectional data hindered our ability to test for mediating factors or the temporality of our associations. Without a longitudinal design, we are unable to test whether insulin resistance precedes frailty and cognitive decline or examine whether subjects transition between body composition categories as they age. Second, our cognitive functioning variable was based on a single measure which did not take into account multiple aspects of cognition. Third, the use of imputed data on measures for muscle mass could be considered a limitation even though this is the procedure recommended by NCHS. Fourth, inflammation and insulin resistance are measured at only one point in time and may vary over time. Despite these limitations, the present study is strengthened by the use of reliable techniques for measuring body composition, the use of biomarkers to measure insulin resistance and inflammation, inclusion of a large representative random sample, and the use of appropriate sample weights and procedures during analysis.

The current study provides preliminary evidence to support the hypothesis that insulin resistance is an important mediator to consider in the association between sarcopenia, obesity, and cognitive functioning. With the doubling of obesity prevalence over the last two to three decades, industrialized countries are expected to experience a rise in the incidence of sarcopenic obesity among the elderly. Furthermore, given that both body composition and cognitive functioning have important implications for quality of life, healthcare costs, morbidity, and mortality, it is important to identify the underlying biological mechanisms which may predispose individuals to comorbidities, such as poor cognitive functioning, adverse body composition, and insulin resistance. In moving forward, the use of longitudinal data and the development of appropriate and reliable definitions for these conditions should facilitate our ability to understand the pathophysiology of age-related comorbidities.

## Figures and Tables

**Table 1:** Sample characteristics for subjects by age group.

	Ages 60–69	Ages 70+
Age (years), median	64	76
Sex (female), (%)	53.63	60.23
Race/ethnicity, (%)		
Non-Hispanic white	78.33	83.16
Non-Hispanic black	8.97	6.08
Hispanic	9.45	9.35
Other	3.24	1.41
Education (years), median	12.00	12.00
History of CVD	15.25	24.70
Low physical activity	23.51	30.76
Hypoalbuminemia	3.83	5.11
Hyperhomocysteinemia	4.71	11.20

**Table 2:** Insulin resistance, CRP, and cognitive functioning comparisons by body composition and age group.

Measure	Sarcopenic obese	Nonsarcopenic obese	Sarcopenic nonobese	Reference group
Aged 60–69				
IR_HOMA,_ median	2.72	3.68	1.92	1.89
CRP (mg/L), median	6.40	3.40	1.90	1.40
Cognitive functioning, mean (SD)	49.59 (23.1)	53.61 (18.2)	50.37 (20.3)	54.80 (18.4)
Aged 70+				
IR_HOMA_, median	2.86	3.19	1.89	1.70
CRP (mg/L), median	4.20	3.00	2.60	2.30
Cognitive functioning, mean (SD)	32.92 (15.8)	43.66 (17.7)	38.26 (15.2)	45.88 (15.4)

**Table 3:** Coefficients predicting cognitive functioning for subjects aged 60–69.

	Model 1	Model 2	Model 3
Sarcopenic obese	−0.91 (1.30)	−0.83 (1.30)	−0.07 (1.31)
Solely sarcopenic	1.14 (0.93)	1.06 (0.93)	1.33 (0.93)
Solely obese	0.19 (0.62)	0.44 (0.68)	0.77 (0.64)
Black	−15.71 (0.93)***	−15.71 (0.93)***	−15.52 (0.93)***
Hispanic	−12.03 (0.98)***	−11.98 (0.98)***	−12.19 (0.97)***
Other	−7.52 (1.47)***	−7.39 (1.47)***	−8.61 (1.49)***
Age	−1.08 (0.09)***	−1.07 (0.09)***	−1.06 (0.09)***
Education	2.80 (0.09)***	2.80 (0.09)***	2.74 (0.10)***
Female	9.25 (0.78)***	9.12 (0.79)***	9.48 (0.78)***
Height	0.15 (0.04)***	0.15 (0.04)***	0.15 (0.04)**
History of CVD	−2.24 (0.72)**	−2.21 (0.72)**	−1.96 (0.72)**
Low physical activity	−3.12 (0.62)***	−3.06 (0.62)***	−3.11 (0.62)***
Hypoalbuminemia	−5.85 (1.29)***	−5.58 (1.32)***	−5.30 (1.29)***
Hyperhomocysteinemia	−4.98 (1.15)***	−4.99 (1.15)***	−4.97 (1.15)***
IR_HOMA_		−0.37 (0.39)	
CRP			−1.01 (0.24)***
*R* squared	.472	.472	0.475

*P < .05 **P < .01 ***P < .001.

**Table 4:** Coefficients predicting cognitive functioning for subjects aged 70+.

	Model 1	Model 2	Model 3
Sarcopenic obese	−7.08 (1.28)***	−5.66 (1.28)***	−7.19 (1.28)***
Solely sarcopenic	−4.19 (0.82)***	−4.39 (0.82)***	−4.20 (0.82)***
Solely obese	−1.43 (0.68)*	0.51 (0.73)	−1.63 (0.69)*
Black	−14.33 (1.19)***	−14.23 (1.18)***	−14.40 (1.19)***
Hispanic	−9.19 (1.01)***	−9.56 (1.01)***	−9.33 (1.01)***
Other	−7.35 (2.03)***	−7.40 (2.01)***	−7.47 (2.03)***
Age (75–79)	−3.10 (0.59)***	−2.91 (0.59)***	−3.04 (0.59)***
Age (80–84)	−8.42 (0.68)***	−8.07 (0.67)***	−8.35 (0.68)***
Age (85+)	−12.02 (0.91)***	−12.49 (0.91)***	−12.03 (0.91)***
Education	2.25 (0.08)***	2.21 (0.08)***	2.24 (0.08)***
Female	3.68 (0.75)***	3.04 (0.75)***	3.80 (0.75)***
Height	−0.11 (0.04)**	−0.13 (0.04)**	−0.10 (0.04)*
History of CVD	−2.63 (0.61)***	−2.42 (0.61)***	−2.64 (0.61)***
Low physical activity	−3.11 (0.57)***	−2.83 (0.56)***	−3.14 (0.57)***
Hypoalbuminemia	−4.12 (1.44)**	−4.35 (1.43)**	−4.70 (1.45)**
Hyperhomocysteinemia	−0.11 (0.89)	−0.27 (0.89)	−0.26 (0.90)
IR_HOMA_		−3.02 (0.45)***	
CRP			0.47 (0.24)
*R* squared	.387	.397	0.388

*P < .05 **P < .01 ***P < .001.
